# Development and usability of a hospital standardized ADL ratio (HSAR) for elderly patients with cerebral infarction: a retrospective observational study using administrative claim data from 2012 to 2019 in Japan

**DOI:** 10.1186/s12877-023-03957-4

**Published:** 2023-04-18

**Authors:** Ryo Onishi, Yosuke Hatakeyama, Koki Hirata, Kunichika Matsumoto, Kanako Seto, Yinghui Wu, Takefumi Kitazawa, Tomonori Hasegawa

**Affiliations:** 1 Department of Social Medicine, Toho University School of Medicine, 5-21-16, Omori-Nishi, Ota-Ku, Tokyo, 143-8540 Japan; 2 School of Nursing, Shanghai Jiao Tong University, 800 Dongchuan RD, Minhang District, Shanghai, 200240 China; 3 Department of Nursing, Faculty of Health Sciences, Tokyo Kasei University, 2-15-1, Inariyama, Sayama, 350-1398 Japan

**Keywords:** Cerebral infarction, Activities of daily living, Quality indicator, Administrative data, Japan

## Abstract

**Background:**

Maintenance of activities of daily living (ADL) during acute hospitalization is an important treatment goal, especially for elderly inpatients with diseases that often leave disabilities, such as cerebral infarction. However, studies assessing risk-adjusted ADL changes are limited. In this study, we developed and calculated a hospital standardized ADL ratio (HSAR) using Japanese administrative claims data to measure the quality of hospitalization care for patients with cerebral infarction.

**Methods:**

This study was designed as a retrospective observational study using the Japanese administrative claim data from 2012 to 2019. The data of all hospital admissions with a primary diagnosis of cerebral infarction (ICD-10, I63) were used. The HSAR was defined as the ratio of the observed number of ADL maintenance patients to the expected number of ADL maintenance patients multiplied by 100, and ratio of ADL maintenance patients was risk-adjusted using multivariable logistic regression analyses. The c-statistic was used to evaluate the predictive accuracy of the logistic models. Changes in HSARs in each consecutive period were assessed using Spearman’s correlation coefficient.

**Results:**

A total of 36,401 patients from 22 hospitals were included in this study. All variables used in the analyses were associated with ADL maintenance, and evaluations using the HSAR model showed predictive ability with c-statistics (area under the curve, 0.89; 95% confidence interval, 0.88–0.89).

**Conclusions:**

The findings indicated a need to support hospitals with a low HSAR because hospitals with high/low HSAR were likely to produce the same results in the subsequent periods. HSAR can be used as a new quality indicator of in-hospital care and may contribute to the assessment and improvement of the quality of care.

**Supplementary Information:**

The online version contains supplementary material available at 10.1186/s12877-023-03957-4.

## Background

In the past few decades, developed countries have faced similar challenges related to the quality and efficacy of care for rapidly aging populations, including an increasing prevalence of chronic illnesses, rising healthcare expenditure, and maldistribution of medical resources. The increasing demand for healthcare services in super-aged society requires overall optimization of effective use of healthcare resources. A reduction in the length of hospital stay (LOS) among patients is one aspect of this strategy. In Japan, which has a super-aged society, the shift from hospital care to community care, including shortening of the LOS, has been promoted as a health policy based on the Community-based Integrated Care System [1–5].

It is important to discharge elderly patients admitted for acute diseases in a condition that allows them to be independent. However, policymakers and physicians were concerned that policies targeting shortening of the LOS and reduction of readmission might have unintended consequences that adversely affect patient care, potentially leading to increased deterioration in the patient’s condition after discharge [6]. For instance, the financial demerit imposed by the Diagnostic Procedure Combination (DPC)/Per-Diem Payment System (PDPS) may have inadvertently pushed some hospitals to avoid readmissions with the same disease, potentially diverted hospital resources and efforts away from other quality improvement initiatives, or worsened the quality of care at resource-poor hospitals. The DPC/PDPS is a Japanese reimbursement system for acute care hospitals introduced in 2003, and the DPC/PDPS database is a national administrative claims and discharge abstract database for inpatient acute care [7–9].

Cerebral infarction is a common disease among the elderly and is responsible for a decline in ADL, particularly manifesting in the form of motor, somatosensory, language, visual, attention, and memory deficits [10]. In Japan, there were a total of 76.0 thousand and 51.0 thousand outpatients with cerebral infarction in 2020, and cerebrovascular disease is the second leading cause of disability after dementia in 2019 [11, 12]. Elderly patients have a high probability of undergoing prolonged hospitalization before discharge; therefore, care during hospitalization, including appropriate discharge management and support, is more important for elderly patients than for younger patients.

Optimal care during hospitalization may reduce worsening of patient outcomes. However, adjustment of patient risks is required to compare and evaluate hospitals with different capacities and functions and ascertain whether each hospital’s care brought standard outcomes. In this regard, risk-adjusted tools or quality indicators, such as the standardized mortality ratio, were used worldwide [13]. Some of them have also been applied to health policy decisions. Administrative data, such as DPC/PDPS data, are useful for designing health policies for disease management, and for the analysis of healthcare processes and patient outcomes [14–19]. In Japan, acute care hospitals can analyze their own quality of care using DPC/PDPS data. In recent years, risk adjustment methods and quality indicators have been actively researched and utilized in clinical practice such as hospitalization decision or patient flow management [13, 20, 21].

This study aimed to develop and evaluate the hospital-level risk-adjusted ADL maintenance during hospitalization for elderly patients with cerebral infarction.

## Methods

### Study design and overview

This retrospective observational study was conducted using DPC/PDPS data. We developed methods for determining the risk-adjusted patients’ risk of a decline in ADL during hospitalization on the basis of previous studies and calculated the HSAR using these methods [22, 23]. In this study, we used anonymized DPC/PDPS data from the Medi-Target benchmarking project managed by the All Japan Hospital Association, which is one of the largest nationwide hospital associations in Japan. The benchmarking project used clinical indicators to improve the quality of hospital care based on DPC/PDPS data. Participation in the project was optional, and the project had 60 participating hospitals in 2020 [24]. This study was conducted in accordance with the Ethical Guidelines for Medical and Biological Research Involving Human Subjects, and was judged as not applicable for ethical review by the Ethics Committee of Faculty of Medicine, Toho University (No. A20067).

### Data and model

Patient data were eligible for enrollment in this study if the patients were at least 65 years of age and had presented to the emergency room with cerebral infarction in fiscal years 2012–2019. In risk adjustment, it is difficult to cover more than one disease. On DPC/PDPS system, cerebral infarction existed as a single claim category and had a severity variable. Since the main objective of this study was to develop a new indicator, a single disease was first included. Only hospitals that provided data for all years from 2012 to 2019 and had treated at least 100 elderly patients with cerebral infarction in each period (2012–2013, 2014–2015, 2016–2017, 2018–2019) were included.

We constructed two HSAR models: an HSAR model using all-period data and the other model using data for each period separately. All inpatients with a primary diagnosis of cerebral infarction were identified using the 10th revision of the International Statistical Classification of Diseases and Related Health Problems (ICD-10) code I63. The HSAR was defined as the ratio of the observed number of patients showing ADL maintenance aggregated at the hospital level to the expected number of patients showing ADL maintenance aggregated at the hospital level multiplied by 100. The Barthel Index (BI) was used for ADL evaluations. Cerebral infarction is likely to cause multiple disabilities associated with declining ADL; therefore, we thought that the BI was appropriate because it is an international index used in hospitals and nursing homes to comprehensively assess ADL. In the BI, ten variables describing ADL and mobility are scored, with a higher number reflecting greater ability to function independently following hospital discharge [25–28]. The BI categorization was performed as follows: BI scores < 60 indicated the need for almost-complete or complete assistance, 60 ≤ BI score < 85 indicated the need for partial assistance, and BI score ≥ 85 indicated independence. In this study, maintenance including improvement of BI was defined as follows: 1) BI score < 85 at admission to BI score ≥ 60 at discharge or 2) BI score ≥ 85 at admission to BI score ≥ 85 at discharge. In this study, maintenance was defined as discharge from the hospital in an independent or partially independent state, rather than a change in the BI score itself. However, going from independent to partially independent is deteriorating. Going from not independent to at least partially independent or from partially independent to independent is considered an improvement, but is included in maintenance.

Multivariable logistic regression analysis was performed to predict the chances of ADL maintenance for each patient with patient-level risk factors (covariates). Coefficients derived from the logistic regression analysis were used to calculate the probability of ADL maintenance. The sum of the predicted probabilities of ADL maintenance (range, 0–1) indicated the total number of patients expected to show ADL maintenance in each hospital. The ratio of the observed number of patients showing ADL maintenance to the expected number of patients showing ADL maintenance served as the standardized ratio for the hospital of interest. An HSAR above 100 indicated that the ADL maintenance ratio was higher than the overall average.

$$\mathrm{HSAR}=\;\left(\frac{\sum\mathrm{Observed}\;\mathrm{number}\;\mathrm{of}\;\mathrm{ADL}\;\mathrm{maintenance}\;\mathrm{patients}}{\sum\mathrm{Expected}\;\mathrm{number}\;\mathrm{of}\;\mathrm{ADL}\;\mathrm{maintenance}\;\mathrm{patients}}\right)\;\times\;100$$

#### Statistical analyses

We calculated hospital performance as an outcome during the study period while considering patient-level case-mix variables to account for the patient’s state at admission. First, we considered the differences in the characteristics between patients showing ADL maintenance and those showing a decline in ADL. Second, in the logistic regression analysis, the data included eight variables: Age, Sex, Surgery during hospitalization, Charlson Comorbidity Index (CCI), Pre-stroke Rankin Scale (PRS) score, Japan Coma Scale (JCS) score, Timing of stroke symptom, and Rresidence before admission. Among these risk-adjusted variables, Age, Sex, Surgery, PRS score, and CCI have been used for risk adjustment in previous studies [18, 29], and the JCS score and Timing of stroke symptom served as severity variables to determine the treatment cost in the DPC/PDPS. For individual variables, age (65 + years) was included in the model as a continuous variable. CCI is an indicator that reflects comorbidities contributing to the deterioration of the patient’s condition and is evaluated by obtaining the sum of the scores for different comorbidities, with higher scores indicating a higher risk of deterioration of the condition [30–32]. In this study, the CCI was based on secondary ICD-10 codes in the DPC/PDPS, and was calculated with Quan’s modification [33]. The calculated CCI values were classified into three categories: i) 0; ii) 1–2; and iii) ≥ 3. The PRS score was categorized as 0–2 (asymptomatic and low symptoms) or 3–5 (moderate and high symptoms). The JCS is the most widely used clinical tool to assess the consciousness level in Japanese acute care [34], and the JCS scores were categorized as 0–3 (disoriented: awake without stimulation), 10–30 (somnolent: responds only with stimulation), or 100–300 (comatose: unarousable despite stimulation) [35]. The timing of stroke symptom was categorized as i) within 3 days or ii) day 4 and later. The categories of PRS and JCS scores and the Timing of stroke symptom defined the severity of cerebral infarction in the DPC/PDPS [36]. Residence before admission was categorized as i) home; ii) hospitalization; or iii) nursing home. This variable was used as a proxy for the patient’s living environment.

To evaluate the predictive accuracy of the logistic models, we used the c-statistic, which was derived by calculating the proportion of concordant pairs. A c-statistic value of 0.5 indicates that the model is no better than random chance in predicting ADL maintenance, and a value of 1.0 indicates perfect discrimination. In this study, Spearman’s correlation coefficient analyses were used to evaluate the relationships between the HSARs for each period because the small sample size did not allow for a normal distribution of the HSAR. As an exploratory analysis, a sensitivity analyses were conducted, excluding the patients with BI score < 60 at admission and the discharged to another hospital, respectively, which could affect the results. For confirmation of risk-adjusted control variables, a two-sided *P*-value < 0.01 was considered statistically significant. In the logistic regression analyses, we obtained the odds ratio and 95% confidence interval (CI), and control variables with two-sided *P*-value < 0.05 were considered to be statistically significant. All statistical analyses were performed using IBM SPSS version 27.

## Results

### Study sample

From April 2012 to March 2020, 36,401 patients (21,293 in the ADL maintenance group and 15,108 in the ADL decline group) at 22 hospitals were included in this investigation. The demographic characteristics of the patients are presented in Table 1. The median age was 79.0 years, and the percentage of male patients was 54.6%. During the study period, 9.2% of the patients underwent surgery during hospitalization. Diabetes without chronic complications was the most common comorbidity and was observed in 8,362 patients (23.0%). Other associated comorbidities included hemiplegia or paraplegia in 3,259 patients (9.0%), dementia in 2,754 patients (7.6%), and peptic ulcer disease in 2,554 patients (7.0%). The demographic characteristics for each period are shown in Table S1 (Supplementary Appendix).

**Table 1:** Demographic characteristics

Characteristics		Total (*n* = 36,401)	Maintenance of ADLs (*n* = 21,293)	Decline of ADLs (*n* = 15,108)	*P* values*
Age	median (IQR)	79.0 (73.0–85.0)	76.0 (71.0–82.0)	83.0 (77.0–88.0)	< 0.001
Male sex	n (%)	19,881 (54.6)	12,848 (60.3)	7,033 (35.4)	< 0.001
Surgery	n (%)	3,339 (9.2)	1,230 (5.8)	2,109 (14.0)	< 0.001
CCI	n (%)				< 0.001
Score 0		25,685 (70.6)	16,291 (76.5)	9,393 (62.2)	
Score 1–2		9,247 (25.4)	4,468 (21.0)	4,779 (31.6)	
Score 3 +		1,469 (4.0)	534 (2.5)	935 (6.2)	
PRS	n (%)				< 0.001
0–2		26,814 (73.7)	18,602 (87.4)	8,212 (54.5)	
3–5		9,587 (26.3)	2,691 (12.6)	6,896 (45.6)	
JCS	n (%)				< 0.001
1–3		3,2861 (90.3)	20,659 (97.0)	12,202 (80.8)	
10 +		3,540 (9.7)	634 (3.0)	2,906 (19.2)	
Timing of stroke symptom	n (%)				< 0.001
Within 3 days		33,970 (93.3)	19,767 (92.8)	14,203 (94.0)	
After 4 days		2,431 (6.7)	1,526 (7.2)	905 (6.0)	
Residence before admission	n (%)				< 0.001
Home		33,280 (91.4)	20,785 (97.6)	12,495 (82.7)	
Hospital		843 (2.3)	252 (1.2)	591 (3.9)	
Nursing home		2,278 (6.3)	256 (1.2)	2,022 (13.4)	
BI at admission	mean ± SD	45.7 ± 38.4	65.3 ± 32.8	18.1 ± 27.1	< 0.001
Comorbidity	n (%)				
Myocardial infarction		394 (1.1)	230 (1.1)	164 (1.1)	
Congestive heart failure		2.089 (5.7)	858 (4.0)	1,231 (8.1)	
Peripheral vascular disease		311 (0.9)	188 (0.9)	123 (0.8)	
Dementia		2,754 (7.6)	776 (3.6)	1,978 (13.1)	
Chronic pulmonary disease		875 (2.4)	498 (2.3)	377 (2.5)	
Rheumatic disease		32 (0.1)	20 (0.1)	12 (0.1)	
Peptic ulcer disease		2,554 (7.0)	1,703 (8.0)	851 (5.6)	
Mild liver disease		483 (1.3)	294 (1.4)	189 (1.3)	
Diabetes without chronic complication		8,362 (23.0)	5,125 (24.1)	3,237 (21.4)	
Diabetes with chronic complication		741 (2.0)	427 (2.0)	314 (2.1)	
Hemiplegia or paraplegia		3,259 (9.0)	1,597 (7.5)	1,662 (11.0)	
Renal disease		782 (2.1)	382 (1.8)	400 (2.6)	
Any malignancy		1,227 (3.4)	693 (3.3)	534 (3.5)	
Moderate or severe liver disease		11 (0.03)	3 (0.01)	8 (0.05)	
Metastatic solid tumor		100 (0.3)	45 (0.2)	55 (0.4)	
HIV		0 (0.0)	0 (0.0)	0 (0.0)	

*n* number of patients, *CCI* Charlson Comorbidity Index, *PRS* Pre-stroke Rankin Scale, *JCS* Japan Coma Scale, *BI* Barthel Index, *IQR* Interquartile range, *SD* standard deviation, *P* values two-sided significance

^*^Patient characteristics were compared using chi-square tests for categorical variables and Mann–Whitney U test for continuous variable (Age and BI at admission)

### HSAR

Chi-square tests and Mann-Whitney U tests showed that all risk-adjusted variables were associated with differences in the changes in ADL (ADL maintenance or ADL decline) (Table 1). Table 2 presents the results of the coefficients and the significance of the logistic regression analysis. All variables showed a significant relationship with differences in the changes in ADL. The results of the coefficients and the significance of logistic regression analyses using data from each period were similar to those for the entire period (Table S2). The results for the c-statistic showed predictive abilities of 0.89 (95% CI, 0.88–0.89) using all-period data, and 0.90 (95% CI, 0.89–0.91), 0.88 (95% CI, 0.88–0.89), 0.89 (95% CI, 0.88–0.89), 0.88 (95% CI, 0.87–0.88) using data for 2012–2013, 2014–2015, 2016–2017, and 2018–2019, respectively. The results of the sensitivity analyses showed in Table S3.

**Table 2:** Variables in the logistic regression analysis

Variables	OR	(95%CI)	*P* values
Age	0.94	(0.94–0.95)	< 0.001
Male sex	1.04	(0.979–1.10)	0.213
Surgery	0.52	(0.32–0.47)	< 0.001
CCI score 0 (reference)			
CCI score 1–2	0.79	(0.74–0.84)	< 0.001
CCI score 3 +	0.59	(0.51–0.68)	< 0.001
PRS 0–2 (reference)			
PRS 3–5	0.38	(0.36–0.41)	< 0.001
JCS 1–3 (reference)			
JCS 10 +	0.45	(0.40–0.50)	< 0.001
Timing of stroke symptom after 4 days (reference)			
Timing of stroke symptom within 3 days	1.15	(1.03–1.29)	0.017
Admission from home (reference)			
Admission from hospital	0.56	(0.46–0.67)	< 0.001
Admission from nursing home	0.29	(0.25–0.34)	< 0.001
BI at admission	1.04	(1.04–1.04)	< 0.001

*CCI* Charlson comorbidity index, *PRS* Pre-stroke Rankin Scale, *CCI* Charlson comorbidity index, *JCS* Japan Coma Scale, *BI* Barthel Index, *OR* Odds ratio, *P values* two-sided significance

**Fig. 1:**
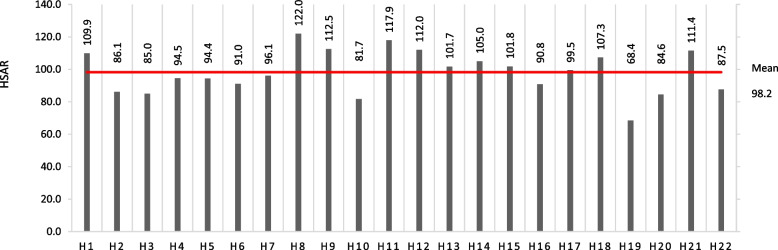
HSAR in each hospital. HSAR = Hospital Standardized ADL Ratio. H = Hospitals.  : mean HSAR

Table 3 shows the mean (± SD) of the HSARs, which were stable in each period and ranged from 83.7 ± 11.6 in 2014–2015 to 101.2 ± 18.9 in 2018–2019. For the whole-period analysis, the mean HSAR (± SD) was 98.2 ± 13.0 (Table 3), and the HSAR in each hospital ranged from 68.4 to 122.0 (Fig. 1). The percentage of hospitals with HSAR over 100 increased from 36.4% in 2012–2013 to 45.5% in 2018–2019. HSAR varied widely across hospitals, and the relationship between the expected and actual numbers of patients showing ADL maintenance is shown in Figure S1. Correlation analyses revealed a significant positive relationship between the changes in HSARs in each consecutive period (Table S4). High/low HSAR hospitals tended to continue their HSAR. Changes in the HSAR by period for each hospital are shown in Figure S2.

**Table 3:** Mean and SD of HSARs

Period	Mean	SD
2012–2013	97.0	11.4
2014–2015	83.7	11.6
2016–2017	99.7	16.1
2018–2019	101.2	18.9
2012–2019	98.2	13.0

*SD* standard deviation, *HSAR* Hospital Standardized ADL Ratio

## Discussion

In this retrospective observational study performed with administrative claims data, the HSAR for cerebral infarction, which was a patient-risk-adjusted indicator with high predictivity, could capture the quality of hospital care. Therefore, we considered the HSAR to be a useful quality indicator to measure the care during acute hospitalization when considering the post-hospitalization life of the elderly. In Japan, the DPC/PDPS is a standard reimbursement system for acute-care hospitals; therefore, the HSAR methods can be widely applied. The results of this study showed that HSAR-based evaluations provide a robust model, and HSAR can be used as a relatively simple tool by hospitals.

Among individual control variables, aging is well known to contribute to the aggravation of cerebral infarction and decline in ADL. The result of chi-square tests and Mann-Whitney U tests for the relationship between changes in ADL and the variables showed significance. After adjusting for patient risk, some hospitals were found to have lower HSAR scores. Since the aim of this study was to identify hospitals that did not meet the standard outcomes for which they should intervene, the HSAR allowed for relative quality assessment and possible improvement on the basis of the case-mix. In this observational study, the confounding variables could not be adjusted; however, the variables used in the regression analysis were considered appropriate. For the risk-adjusted method, we used logistic regression analysis and the case-mix approach as used in previous studies, which were also effective in this study. In the correlation analyses for HSARs, we found that hospitals with high/low HSAR have tend to produce similar results in the following period. Thus, the results suggested that the HSAR for cerebral infarction identified the characteristics of hospital care for ADL maintenance. Notably, we found that hospitals with high/low HSAR were likely to produce the same results in the following period. This result suggested that the HSAR for elderly patients with cerebral infarction was a stable indicator and that the HSAR of the hospitals was influenced by underlying factors. We thought that it is necessary to investigate the characteristics of hospitals with good HSAR so that those with poor HSAR can get beneficial information in improving the ADL care.

Our study included a large sample size to develop risk-adjusted methods for quality indicator. To the best of our knowledge, this is the first large-scale study to calculate the  HSAR using DPC/PDPS data. Importantly, the results for the c-statistics showed predictive ability and the HSAR model was highly robust. However, this study had some limitations. First, the hospitals assessed in this study may not be representative of all hospitals in Japan because they voluntarily participated in the benchmarking project. In addition, the information on hospitals was anonymized, so the bed size and function of hospitals could not be used as a variable, and additional discussion of the results was not possible. We plan a validation study using larger-scale DPC/PDPS data in Japan as a future study. Second, other risk factors for deterioration of ADL, such as medical history or socioeconomic status were not considered in this study [37–39]. The risk of requiring rehabilitation during hospitalization and whether rehabilitation was planned at the time of admission should have been accounted [40]. Third, we could not include the information of discharge destination, which may be a reason for discharging patients in a poor condition, such as those with comorbidities or chronic diseases. This means that if rehabilitation or long-term care is planned at the transfer hospital, the patient might be discharged even if their condition deteriorates after the initial hospitalisation. Other possible adjustment variables such as a Body mass index at admission, but were excluded due to the large number of missing data in the data set used in this study [41, 42]. We would like to incorporate them in an analysis with larger data set.

The results of this study indicated the need for research on the care system for the maintenance of ADL. To improve care during hospitalization, the findings from a good care system of hospitals with HSAR over 100 should be used to evaluate the care in hospitals with HSAR less than 100, and hospitals and policymakers should evaluate the quality of hospital care management, including rehabilitation [43]. In a super-aged society, HSAR based on DPC/PDPS data can be considered a useful indicator for hospital managers and policymakers in healthcare. Future studies should aim to analyze the relationships between HSAR and other quality indicators such as mortality or readmission, and consider other adjustment variables such as socioeconomic status that were not in the DPC/PDPS database [44, 45]. In Japan, the integration of healthcare records and long-term care records is underway, and it is expected that comprehensive care analyses will be possible.

In conclusion, the study of the HSAR as an indicator to support hospital management, including the trends in one's own hospital, may lead to improve the quality of care. In this study, the HSARs for cerebral infarction could be calculated using administrative claims data in Japan. As a new quality indicator, HSARs showed variation among hospitals with comparable case-mixes and might contribute to the development of more useful set of quality indicators for hospitals to improve their quality of care.

## Supplementary Information

**Additional file 1: Table S1.** Demographic characteristics in each period.

**Additional file 2: Table S2.** Variables in the logistic regression analysis in each period.

**Additional file 3: Table S3.** Results of sensitivity analyses.

**Additional file 4: Table S4.** Correlation of HSAR between in each period.

**Additional file 5: Figure S1.** Variation of the expected number of ADL maintenance patients and the actual number of ADL maintenance patients.

**Additional file 6: Figure S2.** Changes of HSAR by fiscal year in each hospital.

## Data Availability

Owing to Japan’s privacy regulations, we are not permitted to share individual-level data. The dataset from this study was held securely in coded form in the AJHA. While legal data-sharing agreements between the AJHA and data providers (hospitals) prohibit AJHA from making the dataset publicly available, access may be granted to those who meet pre-specified criteria for confidential access. External researchers may contact the research team directly in English for assistance with their applications: health@med.toho-u.ac.jp.

## Abbreviations

ADL: Activities of daily living
BI: Barthel Index
CCI: Charlson Comorbidity Index
CI: Confidence interval
DPC/PDPS: Diagnostic Procedure Combination / Per-Diem Payment System
HSAR: Hospital Standardized ADL Ratio
JCS: Japan Coma Scale
LOS: Length of hospital stay
PRS: Pre-stroke Rankin Scale
