# Supplementary material for: Development and usability of a hospital standardized ADL ratio (HSAR) for elderly patients with cerebral infarction: a retrospective observational study using administrative claim data from 2012 to 2019 in Japan
Source: BMC Geriatr. 2023 Apr 18;23:235. doi: 10.1186/s12877-023-03957-4 (PMC10114477; doi:10.1186/s12877-023-03957-4)
Supplement: Supplementary file 5 — Additional file 5: Figure S1. Variation of the expected number of ADL maintenance patients and the actual number of ADL maintenance patients. [file 12877_2023_3957_MOESM5_ESM.pdf]

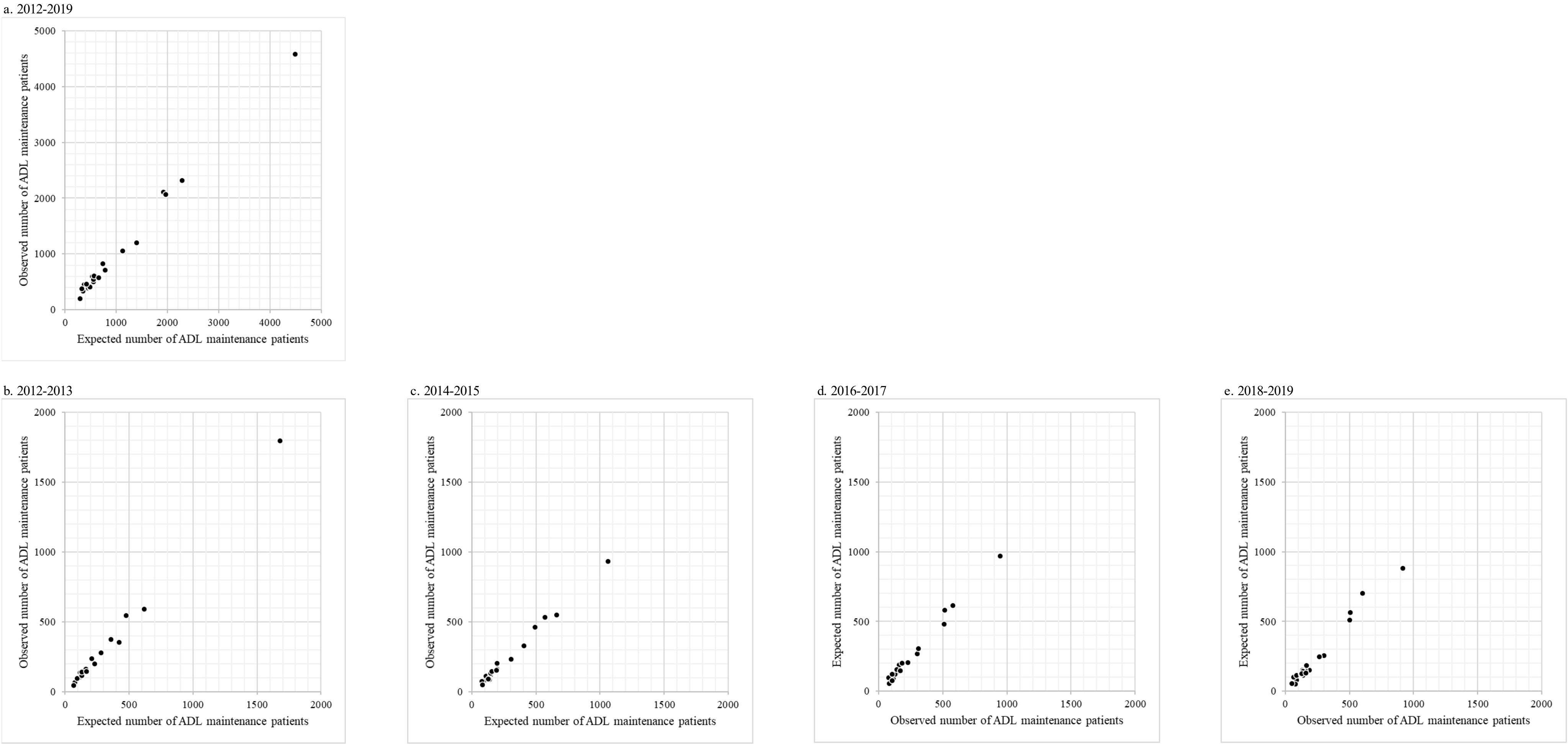

Figure S1. Variation of the expected number of M/I-ADL patients and the actual number of M/I-ADL patients
