# Supplementary figures and images for: Development and usability of a hospital standardized ADL ratio (HSAR) for elderly patients with cerebral infarction: a retrospective observational study using administrative claim data from 2012 to 2019 in Japan
Source: BMC Geriatr. 2023 Apr 18;23:235. doi: 10.1186/s12877-023-03957-4 (PMC10114477; doi:10.1186/s12877-023-03957-4)

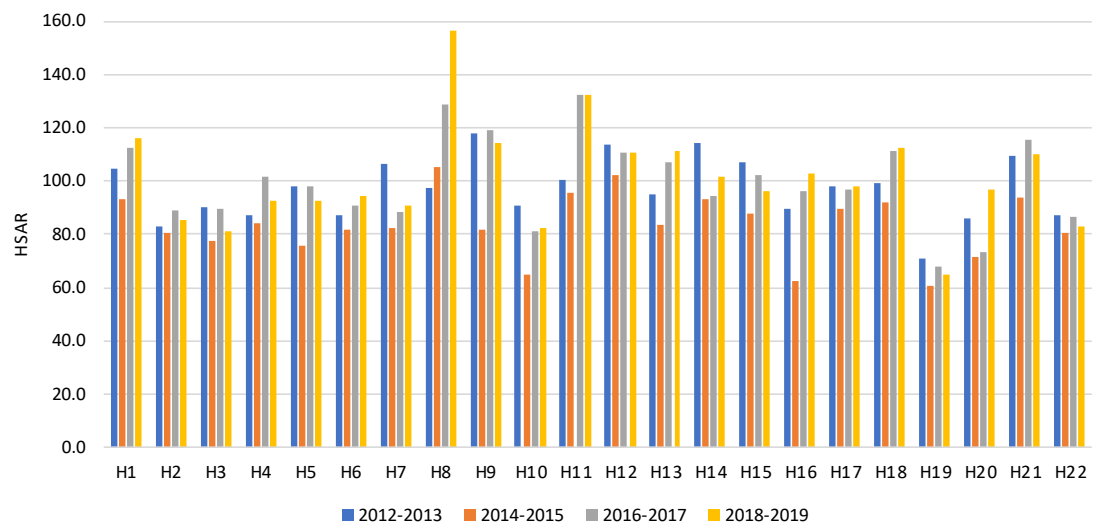

Figure S2. Changes of HSAR by fiscal year in each hospital  
 HSAR = Hospital Standardized ADL Ratio

Supplement: Supplementary file 6 — Additional file 6: Figure S2. Changes of HSAR by fiscal year in each hospital. [file 12877_2023_3957_MOESM6_ESM.pdf]
